# Awake craniotomy for brain tumor resection in the elderly: an institutional experience

**DOI:** 10.1007/s11060-026-05421-w

**Published:** 2026-01-14

**Authors:** Vratko Himic, Victor M. Lu, Roxanne C. Mayrand, Emma R. Sass, Caleigh Roach, Kate Stillman, Sebastian Vargas-George, Jay Chandar, Vaidya Govindarajan, Adham M. Khalafallah, Zachary C. Gersey, Daniel M. Aaronson, Michael E. Ivan, Ashish H. Shah, Ricardo J. Komotar

**Affiliations:** 1 Department of Neurological Surgery, University of Miami Miller School of Medicine, Miami, FL USA; 2 Department of Neurosurgery, Loma Linda University Medical Center, Loma Linda, CA USA; 3 Division of Neurological Surgery, Saint Louis University School of Medicine, St. Louis, MO USA

**Keywords:** Awake craniotomy, Geriatric, Frailty, Safety, Re-admission, Outcomes

## Abstract

**Purpose:**

Awake craniotomy (AC) maximizes safe resection of tumors that encroach on functionally critical areas. However, AC presents additive challenges that are further compounded in the geriatric population. We aim to show that AC is safe and feasible in the elderly, and reveal which peri-operative metrics contribute towards post-operative outcomes, including length of stay, readmissions and discharge disposition.

**Methods:**

We conducted a decade-long retrospective review of AC in patients older than 75 years old. Multivariate linear and logistic regressions were used to identify independent predictors of re-admission, length of stay in ICU and the hospital, and discharge disposition. Variables included Karnofsky Performance Status (KPS), American Society for Anesthesiologists score, frailty index (mFI-11), age in addition to other key metrics.

**Results:**

There were 70 patients with mean age 80 and KPS 75.2 included in our cohort. Glioblastoma was the most common pathology (61.4%) followed by metastasis (22.9%). Only one patient required conversion to general anesthesia, and there were three (4.3%) who had post-operative complications. Re-operation rates following neurosurgical re-admission were 11.4%. Patients with higher pre-operative KPS had shorter hospitalizations (ρ = −0.31, **p = 0.011**). Regarding readmission, mFI-11 was an independent predictor of all-cause 30-day readmission (OR 2.38, **p = 0.007**). In contrast, when restricting analysis to neurosurgery-specific readmissions, age emerged as the only inverse predictor (OR 0.59, **p = 0.010**).

**Conclusions:**

AC is a feasible and necessary tool in the geriatric population. In appropriately organized centers, the surgical success of AC in the elderly can be high. Lower frailty (rather than younger age) predicted shorter stays and reduced all-cause re-admission rates.

## Inroduction

The incidence of central nervous system (CNS) tumors rises with age, reaching 98.22 per 100,000 in those aged 85 years or older [1, 2]. Neuro-oncological surgery in the elderly poses unique challenges due to age-related comorbidities, including reduced organ function, cognitive decline, and slower healing, which complicate standard treatment approaches [3]. A critical consideration is whether these patients can tolerate general anesthesia (GA). Although alternatives to surgical resection, such as stereotactic radiosurgery, chemotherapy, or palliative treatment, are frequently used in the elderly population, surgery remains a central tool [4]. Importantly, whilst age itself is a risk factor, not all elderly patients are co-morbid, and many can tolerate safe and necessary tumor resection

Awake craniotomy (AC) maximizes safe resection, particularly for gliomas, while preserving eloquent brain areas responsible for language, cognition and motor function [5–8]. Compared to carrying out the entire procedure asleep, AC is associated with improved extent of resection (EOR), progression free survival (PFS) and overall survival (OS) [9, 10]. Additionally, patient-reported experiences are favorable; most tolerate AC well, rarely report pain, and often report prefer it over prior procedures under GA [11, 12].

Accurate functional status assessment is central to geriatric medicine. Both the American Society for Anesthesiologists (ASA) physical status classification system and the Karnofsky Performance Status (KPS) are standard medical tools used to measure a patient’s functional ability for pre-operative and neuro-oncological assessment [13, 14]. Additionally, the modified 11-factor frailty index (mFI-11) is a measure of frailty in geriatric patients and has been utilized for the creation of predictive models of surgical complications [15]. These tools are used widely to determine the appropriateness and the likelihood of success of tumor resection. In the geriatric population, features such as the length of stay in the ICU, the hospital, and re-admission rates (both medical and neurosurgical) are key factors to consider; these metrics can have a meaningful physical and psychosocial impact on an elderly patient’s last few months or years of life. Indeed, in this population, difficult conversations with both patient and family are common, particularly about the trade-offs between functional status and length of life; in short, the quality of life should be a key metric to evaluate in this patient cohort.

We report on our institutional experience using AC in the elderly population (those 75 years old and older), focusing on functional status, operative complications, mean length of stay (LOS) in hospital, disposition destination, re-admissions or emergency department presentations, and standard post-operative outcomes of progression-free survival (PFS) and overall survival (OS).

## Methods

### Patient selection and data collection

We conducted a single-institution retrospective cohort study of elderly patients (75 years or older) who underwent AC in the decade up to 2024. This study was approved by our Institutional Review Board (#20160437) at the University of Miami. Demographic variables included sex, age and race. Functional independence was recorded, and frailty was quantified using the 11-item modified Frailty Index (mFI-11). Comorbidities analyzed included congestive heart failure, chronic obstructive pulmonary disease, diabetes mellitus, hypertension, history of cerebrovascular accident, transient ischemic attack, myocardial infarction, percutaneous coronary intervention, peripheral vascular disease, impaired sensorium and tobacco use. Baseline performance status was measured using KPS and ASA class. ASA class, KPS and frailty scores were compiled from patient chart documentation at the time of the procedure, making the collection current and removing the risk of re-interpretation of retrospectively collected data. All procedures were performed as awake craniotomies using an asleep-awake-asleep anesthesia protocol unless aborted. A combination of electromyography, somatosensory evoked potentials and motor evoked potentials were used across the study with options targeted to best maintain safe resection on a case-by-case basis, using cortical and subcortical mapping [16, 17]. Patients were sedated during craniotomy and bone flap removal, then awakened for neurological testing throughout tumor resection.

### Outcome measures

Primary outcomes included postoperative complications, new neurological deficits, hospital LOS, discharge disposition, and all-cause and neurosurgical thirty-day readmission. Complications were categorized as neurosurgical (e.g., hemorrhage, CSF leak, wound infection) or medical (e.g., pneumonia, cardiac event, venous thromboembolism). New neurological deficits were defined as new or worsened motor, sensory, speech, or cognitive impairments persisting at discharge. LOS was measured from admission to discharge, and discharge disposition was recorded as home, inpatient rehabilitation, skilled nursing facility, hospice, or death. Readmissions were defined as any unplanned hospitalization within thirty days of discharge, with neurosurgical readmissions reported separately. Secondary outcomes included PFS and OS. OS was measured from the date of surgery to death from any cause, with survivors censored at last follow-up. PFS was defined as the time from surgery to radiographic progression on follow-up MRI, with patients censored at the last imaging if no progression occurred [18].

### Statistical analysis

Descriptive statistics were used to summarize demographics, tumor characteristics, and outcomes. Continuous variables were reported as means with standard deviations and ranges, and categorical variables as counts with corresponding proportions. Bivariate associations between continuous variables (age, ASA, KPS, anesthesia duration, and LOS) were assessed using Spearman’s rank correlation. Group comparisons used the Mann-Whitney U test for two-group analyses and the Kruskal-Wallis or chi-squared test for categorical variables. Predictors of LOS and length of ICU stay were assessed using multivariable linear regression, discharge disposition and 30-day readmission (both all-cause and neurosurgery-specific) were evaluated using multivariable logistic regression, and discharge disposition was evaluated using a multivariable ordered logistic regression model. Multivariable models used age, ASA score, pre-operative KPS, mFI-11 score, anesthesia duration, and GBM status. Statistical significance was defined as a two-tailed *p* < 0.05. Statistical analysis was conducted in Python 3.12.5 using *pandas* for data management, *statsmodels* for regression modeling (ordered logistic, logistic, linear, Cox), *numpy* for numerical operations, and *matplotlib* and GraphPad Prism (GraphPad Software, Boston, Massachusetts USA) for figure generation.

## Results

### Patient characteristics and perioperative course

A total of 70 patients underwent AC (mean age 80 years; range 75–88) (Table 1). 30 patients (43%) were female, and the mean pre-operative KPS was 74 (range, 60–90). Frailty scores (mFI-11) ranged from 0 to 5 (median 1), and 28 patients (40%) met criteria for frailty, defined as a score of ≥2. ASA classification was documented in 55 patients; most were class III (*n* = 40, 73%), followed by class II (*n* = 12, 22%), and class IV (*n* = 2, 4%). Preoperative neurological deficits were almost universal, present in 64 patients (92%), with 25 patients (36%) having two or more. Language impairment was the most frequent condition (51%, *n* = 36), followed by motor weakness (29%, *n* = 20), cognitive deficits (29%, *n* = 20), gait or balance issues (17%, *n* = 12), and sensory changes (4%, *n* = 3). Ten patients (14%) had a history of seizures before surgery. Tumor laterality was predominantly left-sided (77%, *n* = 54), most commonly in the frontal (31%, *n* = 22) and temporal (31%, *n* = 22) lobes. Regarding tumor type, 67% were gliomas (*n* = 47), of which 91% (*n* = 43) were high-grade, specifically GBM, which contributed to 61% of the lesions across the entire cohort.

**Table 1:** Baseline characteristics of elderly patients undergoing awake craniotomy

Characteristic	Cohort (n = 70)
Mean age, years (range)	79.8 (75.0–87.9)
Sex (n, %)	
Female	30 (42.9%)
Male	40 (57.1%)
Mean KPS (range)	74.1 (8.0–90.0)
ASA Score (n, %)	
2	12 (17.1%)
3	40 (57.1%)
4	2 (2.9%)
N/A	15 (21.4%)
Preoperative seizure (n, %)	10 (14.3%)
Neurologic deficit (n, %)*	
Motor	20 (28.6%)
Speech	36 (51.4%)
Cognitive	20 (28.6%)
Sensory	4 (5.7%)
Gait	12 (17.1%)
None	6 (8.6%)
Lesion laterality (n, %)	
Left	54 (77.1%)
Right	16 (22.9%)
Lesion location (n, %)	
Frontal	22 (31.4%)
Temporal	22 (31.4%)
Parietal	9 (12.9%)
Temporoparietal	8 (11.4%)
Frontoparietal	7 (10.0%)
Frontotemporal	2 (2.9%)

KPS = Karnofsky Performance Score. *Patient could present with more than 1 deficit. ASA = American Association of Anesthesiologists Baseline demographic and clinical presentation variables for the study cohort, including age, sex, Karnofsky Performance Status (KPS), ASA classification, preoperative neurological deficits, lesion laterality, and tumor location. ^*1*^*KPS = Karnofsky Performance Status; ASA = American Society of Anesthesiologists physical status classification*

### Operative course

AC was completed successfully in 97% (*n* = 68) of patients; one patient had an inadequate neurologic exam preventing complete and accurate mapping, and one patient was unable to tolerate the transition from asleep to awake, with repeated failed attempts due to agitation, leading to the procedure being converted. There were no additional cases of aborted procedures, or events of intraoperative complications or seizures. No further patients were excluded to functional or cognitive limitations. The mean anesthesia duration was 266 minutes (range 174-414 mins) (Table 2). Across the cohort, 79% (*n* = 55) of patients underwent both cortical and subcortical mapping, while 19% (*n* = 13) underwent cortical-only mapping during the awake phase. Language mapping was the most common modality, performed in 25 patients (36%), followed by motor mapping in 17 (24%), and both language and motor regions were mapped in 12 patients (17%).

**Table 2:** Intraoperative, pathologic and postoperative characteristics of elderly patients undergoing awake craniotomy

Characteristic	Cohort (n = 70)
Intraoperative complications (n, %)	0 (0.0%)
Mean time under anesthesia, minutes (range)	265.5 (174.0–414.0)
Area mapped (n, %)	
motor	17 (24.3%)
language	25 (35.7%)
language-motor	12 (17.1%)
Not reported	16 (22.9%)
Pathology (n, %)	
Glioma - GBM	43 (61.4%)
Glioma - non-GBM	4 (5.7%)
Metastasis	16 (22.9%)
Lymphoma	3 (4.3%)
Meningioma	3 (4.3%)
other	1 (1.4%)
Postoperative complication (n, %)	3 (4.3%)
Mean length of stay, days (range)	3.5 (2.0–11.0)
Residual neurological deficit (n, %)	36 (51.4%)
Re-admission (n, %)	21 (30.0%)
Readmitted under neurosurgery (n, %)	8 (38.1%)
Reoperation (n, %)	8 (11.4%)

Summary of intraoperative variables, mapping modalities, and histopathologic diagnoses for the study cohort. Summary of postoperative characteristics includes complications, hospital length of stay, residual neurological deficits, readmissions, reoperations, and mortality in the study cohort

### Postoperative outcomes

From our cohort of 70 patients, only three patients (4.3%) experienced complications during index admission due to transient delirium, wound infection, and postoperative seizure (Table 2), representing a low event rate and corresponding to a formal Landriel-Ibanez score of I, II and I respectively. There were no new significant neurological deficits of a different modality than that to which the patients presented, or perioperative deaths. At discharge, 36 patients (51%) had a residual neurological deficit (defined as remaining with remaining deficit in the function that was previously affected, i.e. a patient with weakness remained weak and those with language deficits remained with some aphasia, while 34 (49%) were intact or returned to baseline. Seven patients post-operatively (of 10 pre-operatively) remained with some intermittent seizures, which were controlled with medication. Residual deficits were generally mild, and rates did not differ significantly by mapping type (language: 60%, motor: 47%, combined: 42%; χ^2^ = 1.32, *p* = 0.52).

### Anesthesia duration

The mean anesthesia duration was 266 minutes (range 174–414 minutes). Age was not correlated with anesthesia duration (Spearman ρ = 0.08, *p* = 0.54), and operative time did not differ significantly by mapping type: motor, language, or combined motor-language (Kruskal-Wallis H = 0.45, *p* = 0.80) (Table 2). Patients with residual neurological deficits (*n* = 26) had somewhat longer anesthesia times than those without deficits (278 vs. 254 minutes) (*U* = 283.5, *p* = 0.117). Similarly, readmitted patients (*n* = 15) had longer mean anesthesia times than non-readmitted patients (274 vs. 262 minutes) (*U* = 238.5, *p* = 0.249), although both were not significant.

### Length of ICU stay

**Fig. 1:**
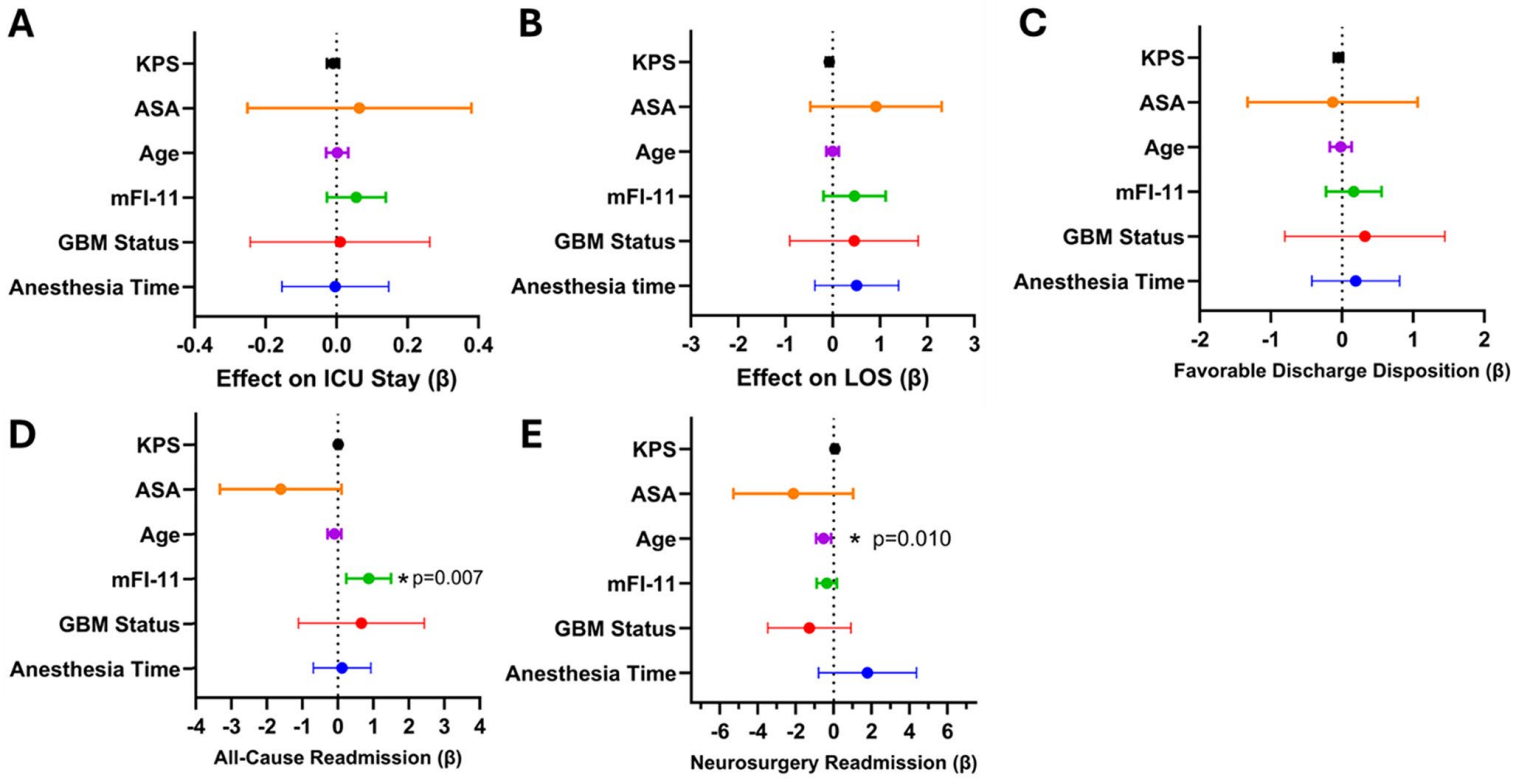
Predictors of post-operative outcomes in geriatric patients after AC. Forest plots demonstrate the correlation coefficient (β) plotted alongside its 95% confidence interval. **A**: multivariate linear regression-derived correlation showing predictors of ICU length of stay. **B**: multivariate linear regression-derived correlation showing predictors of ICU length of stay. **C**: multivariate ordered logistic regression-derived correlation showing predictors of favorable (i.e. home-based, rather than non-home) discharge disposition. **D**: multivariate logistic regression-derived correlation showing predictors of all-cause re-admission in the 30-day post-operative period. Higher mFI-11 scores independently predict higher likelihood of all-cause readmission (*p* = 0.007). **E**: multivariate logistic regression-derived correlation showing predictors of re-admission to the neurosurgical service in the 30-day post-operative period. Higher age independently (and inversely) correlates with neurosurgery service re-admission (*p* = 0.010). These models included frailty (mFI11), age, ASA score, pre-operative KPS, time under anesthesia (mins), and GBM status

On multivariable linear regression analysis, none of the covariates (those being age, ASA, pre-operative KPS, time under anesthesia, frailty (mFI-11 score) or pathology type) demonstrated a statistically significant association with time spent in the ICU post-operatively (Fig. 1A, Table 3

**Table 3:** Predictors of length of hospital and ICU stay

Predictors of Length of Hospital Stay	β	SE	95% CI low	95% CI high	p-value
mFI-11	0.464	0.336	−0.194	1.123	0.167
Age	0.001	0.067	−0.131	0.133	0.989
ASA score	0.916	0.709	−0.473	2.305	0.196
KPS	−0.071	0.037	−0.143	0.001	0.052
Anesthesia time	0.509	0.454	−0.38	1.398	0.262
GBM Status	0.455	0.694	−0.905	1.815	0.512
**Predictors of Length of Stay in the ICU**	**β**	**SE**	**CI low**	**CI high**	**p-value**
mFI-11	0.056	0.042	−0.027	0.139	0.185
Age	0.002	0.016	−0.029	0.033	0.892
ASA score	0.064	0.161	−0.251	0.38	0.689
KPS	−0.01	0.009	−0.027	0.007	0.235
Anesthesia Time	−0.004	0.077	−0.154	0.147	0.963
GBM Status	0.01	0.129	−0.243	0.263	0.938

This table demonstrates the results of multivariate linear regression analysis to evaluate the different independent contributors to length of hospital as well as ICU stay duration during the index admission period. The model included age, ASA score, pre-operative KPS, time under anesthesia, frailty (mFI-11), and GBM status. None of these variables were independent predictors of length of stay. Even though the frailty coefficient in the multivariable LOS model did not reach statistical significance, the adjusted predictions show a roughly +1 extra hospital day for each 2-point increase in mFI-11. Likewise, ICU time increases stepwise with frailty; about 12% more ICU minutes for each 2-point rise in mFI-11

). However, we did find that time spent in ICU did increase in a stepwise manner; for each 2-point increase in mFI-11 score, time spent in the ICU increased by 12%.

### Hospital length of stay

The average hospital LOS was 3.5 days (range 2–11), with most patients discharged within 2–4 days and a minority requiring extended hospitalization. Age showed no association with LOS (Spearman’s ρ = 0.07, *p* = 0.57), with substantial variability in LOS across all age groups, with no clear trend toward longer hospitalizations in the oldest patients (Table 3). In bivariate analysis, preoperative KPS was inversely correlated with LOS (*p* = −0.31, **p = 0.011**), indicating shorter LOS in patients with a higher KPS. No significant relationships were observed with ASA, anesthesia duration, re-admission, functional area being mapped or residual neurological deficit in bivariate analysis. On multivariable linear regression, no covariate reached statistical significance; however, there were suggestive trends: KPS exhibited a trend toward shorter LOS with better functional status (β=–0.071, 95%CI:–0.14–0.00, *p* = 0.052) (Fig. 1B). Even though frailty did not reach statistical significance, the adjusted predictions demonstrated that for each 2-point increase in mFI-11, the LOS was extended by roughly one day.

### Discharge disposition

In terms of discharge disposition, 39 patients (56%) were discharged home (of which 75% without home health services/independent), 23 (33%) to inpatient rehabilitation, and 5 (7%) to skilled nursing or acute care facilities. Higher frailty and longer anesthesia time trended toward less favorable discharge (i.e. higher likelihood of being discharged to a specialist/non-home service), while higher pre-operative KPS trended toward more favorable discharge, but none achieved significance (Fig. 1C, Table 4).

**Table 4:** Predictors of hospital readmission (all-cause, as well as neurosurgical) within 30-days, and favorable discharge disposition

All-Cause Hospital Re-Admission	β	SE	CI low	CI high	OR	OR CI low	OR CI high	p-value
mFI-11	0.867	0.323	0.234	1.499	2.379	1.263	4.479	**0.007**
Age	−0.098	0.099	−0.292	0.096	0.907	0.747	1.101	0.322
ASA score	−1.61	0.874	−3.323	0.104	0.2	0.036	1.11	0.066
KPS	0.012	0.032	−0.051	0.074	1.012	0.951	1.076	0.716
Anesthesia Time	0.117	0.414	−0.693	0.928	1.125	0.5	2.53	0.777
GBM Status	0.664	0.903	−1.107	2.435	1.943	0.331	11.412	0.462
**Neurosurgery Re-Admission**	**β**	**SE**	**CI low**	**CI high**	**OR**	**OR CI low**	**OR CI high**	**p-value**
mFI-11	−0.359	0.27	−0.889	0.17	0.698	0.411	1.186	0.184
Age	−0.526	0.203	−0.924	−0.128	0.591	0.397	0.88	**0.010**
ASA score	−2.125	1.613	−5.287	1.037	0.119	0.005	2.82	0.188
KPS	0.065	0.07	−0.072	0.203	1.067	0.93	1.225	0.353
Anesthesia Time	1.786	1.317	−0.795	4.367	5.965	0.452	78.779	0.175
GBM Status	−1.275	1.118	−3.466	0.916	0.279	0.031	2.499	0.254
**Discharge Disposition**	**β**	**SE**	**CI low**	**CI high**	**OR**	**OR CI low**	**OR CI high**	**p-value**
mFI-11	0.165	0.199	−0.225	0.556	1.18	0.798	1.743	0.407
Age	−0.019	0.078	−0.172	0.134	0.981	0.842	1.143	0.805
ASA score	−0.133	0.61	−1.329	1.063	0.876	0.265	2.896	0.828
KPS	−0.049	0.033	−0.115	0.016	0.952	0.892	1.016	0.139
Anesthesia Time	0.191	0.315	−0.426	0.809	1.211	0.653	2.245	0.543
GBM Status	0.321	0.574	−0.805	1.446	1.378	0.447	4.248	0.576

A multivariable logistic regression model was used to identify predictors of hospital readmission and neurosurgery specific readmission after awake craniotomy. Model covariates included frailty (mFI-11), age, ASA score, pre-op KPS, time under anesthesia, and GBM status. mFI-11 scores were an independent predictor for increased chance of re-admission in the 30-days post-operative period for any cause, whereas age was an inverse independent predictor of re-admission to the neurosurgery service in the same period (older patients had lower odds of neurosurgery specific readmission). A multivariable ordered logistic regression was performed to identify predictors of favorable discharge destination after awake craniotomy (defined as any non-home discharge). The model included frailty (mFI11), age, ASA score, pre-operative KPS, time under anesthesia (mins), and GBM status. No covariate reached statistical significance in this model; point estimates suggested expected directions

### Readmissions

30% of patients (*n* = 21) required hospital re-admission within 30 days of discharge for any reason. Of these, eight patients (38% of readmissions) were readmitted under the neurosurgical service. In bivariate analysis, neither age (*p* = 0.85) nor preoperative KPS (*p* = 0.43) were found to be associated with readmission risk. Multivariable logistic regression identified frailty (mFI-11) as the only independent predictor of all-cause readmission: a one-point increase in mFI-11 was associated with 2.38 times higher odds of readmission (β = 0.867, SE = 0.323, OR 2.38, **p = 0.007**) (Fig. 1D, Table 4). In contrast, when restricting analysis to neurosurgery-specific readmissions (*n* = 8), age emerged as the only predictor, and it was an inverse predictor: each additional year was associated with 41% lower odds of readmission to the neurosurgery service in the 30 days post-AC (β = −0.526, SE = 0.203, OR 0.59, **p = 0.010**) (Fig. 1E, Table 4). Frailty was not significant in this subgroup (*p* = 0.18). This shows an interesting differential re-admission pattern for all-cause readmission versus neurosurgery-only readmission.

### Survival, progression and re-operation

When stratified by primary pathology, outcomes diverged early with patients with GBM (*n* = 43) having a median OS of approximately 13.8 months. In contrast, patients with non-GBM tumors (*n* = 27) had a median OS of 41.9 months; as expected, significantly longer than the GBM cohort (log-rank **p = 0.016**). 34 patients (49%) experienced disease progression or death by the time of last follow-up (median: 165.5 days, IQR: 379). Radiographic progression on MRI occurred in 16 patients (23%), and among these, seven (44%) subsequently died. Across the cohort, three patients underwent repeat craniotomy, while two patients underwent needle biopsy with laser tumor ablation.

## Discussion

The utility of AC during intracranial tumor resection remains an important tool, however, remains relatively under-evaluated in geriatric populations [5–7]. Our findings are consistent with prior studies that suggest that AC can be safe and effective in geriatric populations when attention is paid to the patient’s functional status [9, 19]. Almost all (97%) of the patients tolerated AC well, with only two patients having to convert to GA. Our cohort experienced no intraoperative complications, and only three patients (4.3%) experienced complications during the index admission. Our results suggest that preoperative functional status and systemic fitness, rather than age or pathology, most strongly affect patient recovery, as measured by LOS and rate of readmission. A very high rate of tolerance of AC and a mean KPS score of 74 (range, 60–90) align with previous research suggesting that preoperative physical status, as measured by KPS, was the most predictive of whether a patient can tolerate AC [20–22].

We suggest that frailty and functional status, rather than chronological age, are the critical determinants of perioperative outcomes in elderly patients undergoing AC [23, 24]. Frailty (as measured by mFI-11) emerged as the only independent predictor of 30-day all-cause readmission, with each 1-point increase conferring 2.38-fold higher odds of readmission (95%CI:1.26–4.48, **p = 0.007**). Meanwhile, age itself showed no association with all-cause readmission (*p* = 0.85), challenging the use of age-based cutoffs in surgical decision-making [23]. Similarly, preoperative KPS inversely correlated with hospital LOS (*p* = 0.31, **p = 0.011**). In addition, even though frailty did not reach statistical significance in affecting hospital LOS, each 2-point increase in mFI-11 extended the LOS by roughly one day. Likewise, regarding ICU stay, each 2-point increase in mFI-11 score increased ICU time by 12%. These findings reiterate that baseline physiologic reserve and systemic fitness, rather than chronological age, drive short-term recovery trajectories [25, 26]. The substantial effect size of frailty on readmission risk, combined with the observation that 40% of this cohort met frailty criteria (mFI≥2), argues for systematic frailty assessment as a standard component of preoperative risk stratification in elderly neurosurgical candidates.

However, readmission patterns revealed a critical paradox that illuminates the interplay between biological aging and clinical decision-making. While frailty predicted all-cause readmission, age demonstrated an inverse relationship with neurosurgery-specific readmissions, with each additional year decreasing the odds by 41% (OR 0.59, **p = 0.010**). This appears contradictory, given that age and frailty typically correlate, and that both were associated with worse outcomes across other metrics. However, this finding likely reflects treatment selection bias rather than any protective effect of aging. Neurosurgical readmission serves as a proxy for aggressive tumor management: reoperation for recurrence, laser ablation, or further resection. These interventions are more commonly offered to (and accepted by) younger elderly patients (75–80 years) compared to the oldest patients (85+ years), who may receive conservative or palliative management regardless of tumor behavior or functional status [27–29]. This interpretation is supported by the absence of any relationship between age and length of stay (*p* = 0.07 and *p* = 0.57, respectively), suggesting that age exerts no linear effect on immediate perioperative recovery. Caution is warranted when interpreting age-outcome relationships in elderly surgical cohorts, as observed associations may reflect clinical practice patterns as much as biological capacity.

By examining both short-term perioperative metrics and long-term survival as co-primary outcomes, this study advances the ongoing effort to define what constitutes clinical endpoints in geriatric neurosurgery. Hence, it addresses a central tension within geriatric neurosurgery and neuro-oncology related to the bipolar effects of operative intervention [30]. Does extending survival justify perioperative risk, and how can such risks and preservation of independence be adequately assessed as metrics of success? The implications of these findings extend beyond the immediate cohort. Firstly, in our analysis, frailty emerged as the only independent predictor of 30-day readmission, challenging fundamental age-based decision-making in surgery. These results advocate for the prospective development of a neurosurgery-specific frailty index, integrating functional independence, systemic comorbidities, cognitive reserve, and neurologic deficits, to provide a reproducible predictive tool for operative decision-making in the elderly [26, 31, 32]. In a 334-patient cohort of general neurosurgical patients, Adebola and colleagues found that age itself was not a significant predictor of mortality and morbidity. However, the Glasgow Coma Score (GCS) did significantly contribute in addition to traditional frailty tools (mFI-5/mFI-11, ASA). They noted a further paradox in which lower mFI-5 patients still experienced poor outcomes, which suggests that broad frailty measures can misclassify individuals [32]. Additionally, the decision to initiate nonoperative versus operative intervention in elderly patients is an ongoing discussion, as elderly patients with GBM also face a distinct vulnerability to the toxicities of nonoperative oncologic therapies [33].

This study has several limitations. First, as a retrospective, single-center analysis performed at a tertiary medical center, our findings are subject to selection and referral bias, which may limit generalizability to broader populations. The relatively small sample size (*n* = 70) limits statistical power, particularly for subgroup analyses. The heterogeneity of pathology (GBM, non-GBM, meningioma, and metastasis) also contributes to overall reporting difficulty, although disease-specific results are reported. Additionally, follow-up was variable, which limits the ability to consistently evaluate long-term outcomes. In addition, the lack of a control group (for example patients undergoing non-awake general anesthetic surgery) further limits the conclusions we can make; in this paper we focus solely on the awake cohort. A prospective, multi-institution study is warranted to validate these findings and refine perioperative strategies; this would improve the external validity of our work and take into account different awake craniotomy practices (such as patient inclusion criteria), build the power of the study by including larger numbers of each pathology, and improve our ability to quantify subgroup analyses with more reliability.

Our findings add to the evidence that awake craniotomy can be safely and effectively performed in well-selected elderly patients at centers with established experience in awake mapping through both exposure to cases during resident training as well as at the senior fellow level [9, 19]. This is particularly pertinent when conversion to GA is occasionally required, and unsuccessful awake exams, such as the case in our series where the patient could not tolerate AC [34]. These considerations highlight the importance of careful patient selection and experienced teams, but they should not preclude offering awake craniotomy to appropriately chosen older adults.

## Conclusion

AC in geriatric patients was practical and safe with low complication rates. Favorable neurologic outcomes demonstrated that many patients with preoperative deficits improved or returned to baseline by discharge. Interestingly, recovery was determined less by age and more so by functional fitness. A higher pre-operative KPS correlated with shorter LOS. The mFI-11 independently predicted all-cause readmission, suggesting the important role of frailty as a consideration in this cohort. Each two point increase in mFI-11 score trended towards prolonging both the hospital stay, and the ICU stay duration. Our findings suggest that routine frailty and functional assessment should help guide selection and perioperative planning in neuro-oncologic geriatric surgery candidates, and that AC is feasible and safe in the elderly.

## Data Availability

All data supporting the findings of this study are available within the paper, and any further queries can be directed to the corresponding author.

## Declaration

### Statement on relevant guidelines and regulations

all methods were carried out in accordance with relevant guidelines and regulations. This project was approved by our Institutional Review Board (#20160437) at the University of Miami. Informed consent was not sought as it was not necessary in this instance; this work is entirely composed of retrospective review of patient charts with no intervention or additional patient contact.
